# Major amputation rates and outcomes for Aboriginal and Torres Strait Islander and non-Indigenous people in North Queensland Australia between 2000 and 2015

**DOI:** 10.1186/s12902-021-00764-z

**Published:** 2021-05-21

**Authors:** Tejas P. Singh, Joseph V. Moxon, Michael T. Meehan, Rhondda Jones, Yvonne Cadet-James, Jonathan Golledge

**Affiliations:** 1 Queensland Research Centre for Peripheral Vascular Disease, College of Medicine and Dentistry, James Cook University, 4811 Townsville, Queensland Australia; 2 The Department of Vascular and Endovascular Surgery, The Townsville University Hospital, Townsville, Queensland Australia; 3 The Australian Institute of Tropical Health and Medicine, James Cook University, Townsville, Queensland Australia; 4 Anton Breinl Research Centre for Health Systems Strengthening, Australian Institute of Tropical Health and Medicine, James Cook University, Townsville, Queensland Australia

**Keywords:** Diabetes, Lower limb amputation, Peripheral artery disease, Australia

## Abstract

**Background:**

This study estimated the incidence of major amputation for people in North Queensland, Australia, examined changes in amputation rates over time and investigated survival after major amputation.

**Methods:**

This was a retrospective study of patients who underwent a major amputation above the ankle between 2000 and 2015. Major amputation rates and incidence rate ratios (IRR) were calculated using census data to define the at-risk population. Associations between risk factors and calendar year with major amputation were assessed using quasipoisson regression. Kaplan-Meier survival and Cox-proportional hazard analyses estimated the incidence of and risk factors for all-cause mortality.

**Results:**

The annual incidence of major amputation was estimated to be greater in Aboriginal and Torres Strait Islanders than non-Indigenous people (IRR 2.75, 95% CI 1.92 to 3.84). After adjusting for population growth, the annual incidence of major amputations did not change significantly over time for either groups. Aboriginal and Torres Strait Islander people were at greater risk of all-cause mortality after major amputation compared to non-Indigenous people, although this association was not significant after adjusting for other risk factors (hazard ratio 1.24, 95% CI 0.82 to 1.90).

**Conclusions:**

The incidence of major amputation in North Queensland has not reduced over time, indicating the need for better preventative treatments, particularly in Aboriginal and Torres Strait Islander people.

**Supplementary Information:**

The online version contains supplementary material available at 10.1186/s12902-021-00764-z.

## Background

Lower extremity amputations, including excision of toes (usually referred to as minor amputation) and amputations above the ankle (usually referred to as major amputation), are significant complications of diabetes-related peripheral neuropathy and peripheral artery disease (PAD) [1]. Individuals that undergo a major amputation have reduced quality of life and approximately three fold higher risk of mortality than people who have not undergone a major amputation [2]. There is considerable variation in amputation rates in different geographical locations [1]. The reported cumulative age-adjusted incidence of first lower extremity amputations in the United Kingdom ranges from 5 to 176 per 100,000 across different centres [1].

Recent studies have suggested a decline in major amputation rates despite a rise in the prevalence of diabetes [1, 3]. A report from Queensland Australia has suggested a significant 45% decrease in diabetes-related major amputations between 2005 and 2010 [4]. There has been limited study of trends in major amputation rates in high-risk sub-populations, such as Aboriginal and Torres Strait Islander people. Census data suggest that Aboriginal and Torres Strait Islander people represent approximately 3% of the total Australian population [5]. Aboriginal and Torres Strait Islander people more frequently have risk factors for major amputation, such as PAD [6], diabetes, end-stage renal disease [7] and smoking [8]. A study from Western Australia reported that Aboriginal and Torres Strait Islander people with diabetes were approximately 40 times more likely to undergo a major amputation compared to non-Indigenous people with diabetes during the years 2000 to 2008 [9]. Similarly, in a centre in Cairns, Australia, where approximately 10% of the population are Aboriginal or Torres Strait Islander people [10], 52% of major amputations between 1998 and 2008 occurred in this group [11]. Collectively, these data highlight a high incidence of major amputation in Aboriginal and Torres Strait Islander people [12]. There has been no previous study of trends in major amputation rates over time in this population or comparison of clinical outcomes between Aboriginal and Torres Strait Islander people and non-Indigenous people having major amputations. Such data are important in order to assess whether current services for the high-risk foot are achieving their goals. The current study was focused in North Queensland, Australia where the prevalence of diabetes-related foot disease is reported to be high [11, 13]. This study aimed to assess the annual incidence of and trends over time in major amputation for Aboriginal and Torres Strait Islander and non-Indigenous people in North Queensland. The study also aimed to identify significant risk factors for major amputation and compare the survival of Aboriginal and Torres Strait Islander and non-Indigenous people after major amputation.

## Methods

### Design

This was a retrospective study performed by assessing hospital records on all patients who underwent a non-traumatic major amputation at The Townsville Hospital and Health Service (THHS) between 2000 and 2015. The THHS is the main tertiary centre and health service for Northern Queensland. It has a geographical coverage of approximately 148,000 square kilometres and serves approximately 4.8% of the total Queensland population [14]. Approximately 8% of individuals residing in this region are of Aboriginal and Torres Strait Islander descent, which is nearly double the average for Queensland [14]. Ethical approval was granted from the Townsville Hospital and Health Services Ethics Committee (HREC/13/QTHS/125) and this included a patient consent waiver, which was required due to the retrospective design of the study [15, 16]. A 5-member Aboriginal and Torres Strait Islander reference committee from James Cook University was consulted for approval of the research as previously described [13]. All methods were carried out in accordance with relevant guidelines and regulations. Patients who underwent a major amputation (including above the knee amputation [AKA], below the knee amputation [BKA], or hindquarter amputation [HQA]) were included. Patients who underwent these operations were identified from the operating rooms management system (ORMIS). Aboriginal and Torres Strait Islander status was based on self-identification by patients at the time of admission. These data were extracted from hospital records.

### Measures

Clinical characteristics collected for each patient included: age at the time of amputation; sex; history of diabetes, hypertension, PAD, ischaemic heart disease (IHD) and end-stage renal disease (ESRD); and operation type. Hypertension and diabetes were defined by history or medical treatment for these conditions. IHD was defined by a history of myocardial infarction, angina or coronary revascularization. ESRD was defined by requirement for dialysis. PAD was defined by an ankle-brachial index (ABI)<0.9 and/or imaging demonstrating stenosis or occlusion of the lower limb arteries as previously described [14, 15]. Operative data were obtained from the ORMIS. Clinical characteristics and follow-up data were retrieved from out-patient visits, hospital chart reviews and inpatient admissions as previously described [14, 15]. Data from these sources were reviewed by the investigators to ensure their accuracy. The primary outcome recorded from follow-up data was all-cause mortality which was defined to include deaths from any cause. If a patient did not die their follow-up was censored at the date of their last recorded hospital visit. The secondary outcome recorded was the requirement for contralateral major amputation.

### Statistical analysis

The annual, population-standardised incidence of major amputation in relation to Aboriginal and Torres Strait Islander status, age and diabetes status was estimated with the following assumptions about the population serviced by the THHS:

i) It included 217,893 individuals located in Townsville, Charters Towers, Ingham, Cardwell, Ayr, Magnetic and Palm Islands, Home Hill, Hughenden and Richmond [14] based on the 2011 Australian Bureau of Statistics Census [17];
ii) 15,409 individuals were Aboriginal or Torres Strait Islander people and 189,684 were non-Indigenous based on the 2011 Australian Bureau of Statistics Census [17]. Individuals that did not state Aboriginal or Torres Strait Islander Status (*n*=12,800) were re-assigned to the Aboriginal and Torres Strait Islander people or non-Indigenous categories in proportion to the numbers recorded for those who did report Aboriginal or Torres Strait Islander status (i.e. in the ratio 15,409: 189,684 for Aboriginal and Torres Strait Islander and non-Indigenous people, respectively).
iii) The numbers of at-risk individuals (i.e. Aboriginal and Torres Strait Islander and non-Indigenous people with and without diabetes) in different age groups (034; 3544; 4554; >55) were estimated using state-wide diabetes prevalence rates reported by the Australian Bureau of Statistics [18]. The age categories used were chosen to match the age classifications provided by the available state-wide diabetes prevalence rates [18];
iv) Since an estimate of the at-risk population size was only available for the calendar year 2011, the at-risk population for the remaining years in the study period was estimated assuming constant population growth. In particular, estimates of the at-risk population sizes backward in time to the year 2000 and forward in time to the year 2015 were generated assuming a 2% (multiplicative) annual decrease or increase in the baseline 2011 value (in line with the estimated annual population growth rate in the THHS catchment over this period [19]) across all groups.

A reported weakness of the Australian census is an under-estimate of the number of Aboriginal and Torres Strait Islander people (approximately 14.7%, standard error 2.6%) for Queensland [5]. Sensitivity analyses were therefore performed to account for (i) potential errors in the classification of Aboriginal and Torres Strait Islander people in the census data; (ii) a potential census undercount in the total Aboriginal and Torres Strait Islander populations; and (iii) variation in the annual growth rate in the population across the study period. In the first sensitivity analysis, individuals who were not classified in the census (i.e. not specified to be Aboriginal and Torres Strait Islander or non-Indigenous) were considered non-Indigenous. In the second sensitivity analysis, the at-risk Aboriginal and Torres Strait Islander population was increased by 20% to account for a potential census undercount in this group and the unspecified individuals were removed from the analysis [5]. In the final sensitivity analysis the population growth rate was varied between 0.5 and 10% per annum. The annual incidence of major amputation was estimated as the number of crude amputations in each calendar year (numerator) over the size of the estimated at-risk population for the relevant calendar year (denominator) as previously described [4]. Major amputation incidence rates were stratified by Aboriginal and Torres Strait Islander status and were expressed per 100,000. Incidence rate ratios (IRR) were calculated to compare annual major amputation incidence rates between groups. Trends in annual major amputation rates per 100,000 for Aboriginal and Torres Strait Islander and non-Indigenous people were graphically compared with national major amputation rates obtained from the Australasian Vascular Audit (AVA) between 2010 and 2014 [20]. To account for over dispersion in amputation data, quasipoisson regression was used to assess the effect of Aboriginal and Torres Strait Islander status, age, sex, diabetes status and calendar year on the annual incidence of major amputations [21]. The regression model incorporated estimates of the population size in each subcategory as an offset.

Clinical characteristics were compared between Aboriginal and Torres Strait Islander and non-Indigenous people. Histograms, skewness and kurtosis tests suggested that continuous data were not normally distributed. Thus, continuous data were summarized using median values and inter-quartile ranges and compared between groups using the Mann-Whitney U test. Nominal data were presented as count and percent (unless otherwise stated) and compared using chi-squared and Fishers exact tests. Kaplan-Meier analysis was used to examine patterns of all-cause mortality in Aboriginal and Torres Strait Islander and non-Indigenous people, with differences compared using the log rank test. Multivariable Cox proportional hazards analysis was used to assess the association between ethnicity and all-cause mortality, adjusting for relevant confounding variables (age, sex, IHD, diabetes, ESRD and hypertension). These variables were included in the Cox proportional hazard analysis as they are established risk factors for mortality [6]. All Cox regression models presented in this paper were found to conform to the proportional hazards assumption. Analyses were performed using STATA version 16.1 (StataCorp, College Station, Texas, USA) and R version 3.4.4 (www.r-project.org/). *P* values<0.01 were considered statistically significant.

## Results

### Patient characteristics

During the study period, 374 patients underwent a non-traumatic lower limb major amputation. Of these, 70 (18.7%) patients identified as Aboriginal and Torres Strait Islander people and 304 patients identified as non-Indigenous (Table1). Patients identifying as Aboriginal and Torres Strait Islander people were more likely to be female (51.4% vs. 31.9%, *p*=0.002), have diabetes (83.3% vs. 50.4%, *p*<0.001) and ESRD (31.5% vs. 14.7%, *p*=0.003) than non-Indigenous patients. There were no statistically significant differences in the type of major amputation performed in Aboriginal and Torres Strait Islander and non-Indigenous patients.

**Table 1:** Comparison of Aboriginal and Torres Strait Islander and non-Indigenous patients

	Aboriginal and Torres Strait Islander (*n*=70)	Non-Indigenous(*n*=304)	*p*-value
*Characteristics at recruitment*			
Age	56.0 (44.0-65.3)	68.0 (54.0-75.8)	**<0.001**
Males	34 (48.6%)	207 (68.1%)	**0.002**
Diabetes	45 (83.3%)	133 (50.4%)	**<0.001**
Hypertension	36 (66.7%)	172 (65.2%)	0.831
IHD	27 (50.0%)	122 (46.2%)	0.611
PAD	29 (53.7%)	147 (55.5%)	0.100
ESRD	17 (31.5%)	39 (14.7%)	**0.003**
*Outcomes during follow-up*			
Amputation			0.789
AKA	28 (40.0%)	131 (43.1%)	
BKA	42 (60.0%)	172 (56.6%)	
HQA	0 (0%)	1 (0.3%)	
Contralateral amputation	10 (14.3%)	33 (10.9%)	0.417
Death	47 (67.1%)	160 (52.6%)	**0.028**
Follow-up (years)	2.3 (0.45.7)	3.3 (1.38.1)	**0.020**

Continuous data are presented as median [interquartile range] and were compared using Mann-Whitney U test. Nominal data are presented as number (%) and were compared using Pearsons 2 test

*IHD*ischaemic heart disease, *PAD*peripheral artery disease, *ESRD*end-stage renal disease, *AKA*above knee amputation, *BKA*below knee amputation, *HQA*hindquarter amputation

*P*-values highlighted in bold indicate significant differences

### Estimated annual incidence of major amputation

**Fig. 1:**
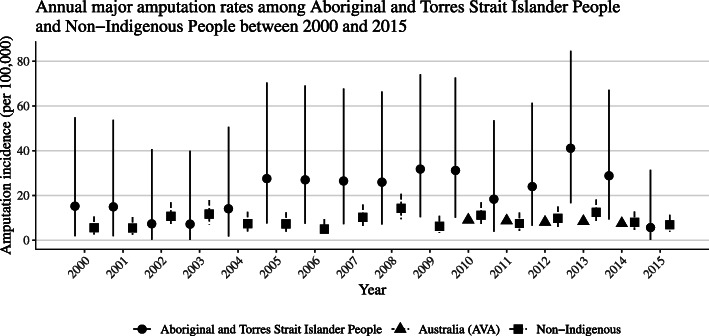
Estimated annual major amputation incidence for Aboriginal and Torres Strait Islander and non-Indigenous people between 2000 and 2015.Incidence presented per 100,000 Aboriginal and Torres Strait Islander and non-Indigenous people using the 2011 census as the at-risk population. The at-risk population denominator was adjusted throughout the study period based on an annual population growth rate of 2%. The circular points represent trends in amputation incidence in Aboriginal and Torres Strait Islander people. The square points represent trends in amputation rates in non-Indigenous people. The triangular points represent national Australian amputation rates (Australasian Vascular Audit [AVA]). Vertical lines represent the 95% CI. For the AVA data financial year estimates are presented

The estimated annual major amputation rates in both Aboriginal and Torres Strait Islander and non-Indigenous people were higher than the previously reported national Australian averages (Fig.1). The incidence of major amputation was higher in Aboriginal and Torres Strait Islander compared with non-Indigenous people (IRR 2.75, 95% confidence interval (CI) 1.92 to 3.84). Diabetes, male sex, ESRD and older age were also associated with a higher rate of amputation (Table2). Sensitivity analyses suggested that the reported associations between amputation and Aboriginal and Torres Strait Islander status, diabetes, ESRD, sex and age were robust (see Supplementary Table1). When it was assumed that there was a static background population (0% annual growth rate) over the 16-year study period a slight increase in amputation incidence over time was estimated (IRR 1.04 95% CI [1.011.07]). This effect was lost when an annual population growth rate of 2% was assumed (IRR 1.02 95% CI [0.991.05]) and only began to reverse once the annual growth rate in the background population was assumed to exceed 5% (see Supplementary Table2).

**Table 2:** Independent risk factors for major amputation identified through quasipoisson regression

Characteristic	IRR (95% CI)	*P*-value
Aboriginal and Torres Strait Islander people	2.75(1.923.84)	**<0.001**
Diabetes	12.77(9.2816.81)	**<0.001**
Male sex	2.00(1.542.61)	**<0.001**
Age 034 years	Reference	
Age 3544 years	3.51(1.846.79)	**<0.001**
Age 4554 years	5.35(3.029.87)	**<0.001**
Age>55 years	11.57(7.0220.29)	**<0.001**

For binary variables, patients that did not have the risk factor were used as the reference group

*CI*confidence interval, *IRR*incidence rate ratio

### All-cause mortality and contralateral major amputation

**Fig. 2:**
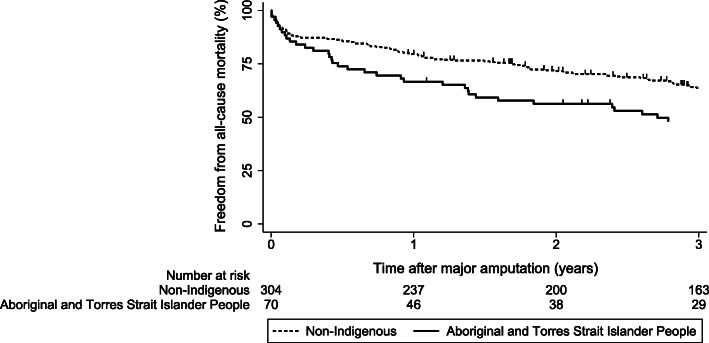
Kaplan-Meier curve illustrating the cumulative proportion of Aboriginal and Torres Strait Islander and non-Indigenous patients with a major amputation who survived.Differences between groups were compared using the log-rank test (*p*=0.005). The thick line represents the cumulative proportion of Aboriginal and Torres Strait Islander people that survived. The interrupted line represents cumulative proportion of non-Indigenous people that survived. Vertical lines represent participants who were censored during follow-up

The median (inter-quartile range) follow-up of Aboriginal and Torres Strait Islander and non-Indigenous patients were 2.3 (0.4 to 5.7) and 3.3 (1.3 to 8.1) years, respectively (*p*=0.020). Kaplan-Meier survival curves illustrating the cumulative proportion of patients with a major amputation who survived are shown in Fig.2. A greater proportion of Aboriginal and Torres Strait Islander patients died during follow-up compared to non-Indigenous patients (56.7% vs. 42.7% at 3 years follow-up). Differences in the cumulative proportions between groups were statistically significant (log-rank test *p*=0.005). In the unadjusted Cox proportional analysis, Aboriginal and Torres Strait Islander patients who underwent a major amputation were at greater risk of all-cause mortality during follow-up compared to non-Indigenous patients (hazard ratio, HR, 1.59, 95% CI 1.14 to 2.20; *p*=0.006). The association remained significant when adjusted for age and sex (HR 1.79, 95% CI 1.28 to 2.51, *p*=0.001) but no longer remained significant when adjusted for age, sex, IHD, ESRD and hypertension (HR 1.24, 95% CI 0.82 to 1.90, *p*=0.309) (Table3). ESRD was significantly associated with a greater risk of all-cause mortality in the adjusted analysis (HR 1.90, 95% CI 1.30 to 2.77, *p*=0.001). No significant difference in the incidence of contralateral major amputation was observed between Aboriginal and Torres Strait Islander or non-Indigenous patients (14.3% vs. 10.9% respectively, *p*=0.417, Table1 and Supplementary Fig.1).

**Table 3:** Cox proportional hazard analyses for the association between participant characteristics and all-cause mortality

	Unadjusted HR (95%CI)	*P*-value	Adjusted HR^a^ (95%CI)	*P*-value
Death				
Aboriginal and Torres Strait Islander people	1.59 (1.142.20)	**0.006**	1.24 (0.821.90)	0.309
Age	1.02 (1.011.03)	**<0.001**	1.03 (1.011.04)	**<0.001**
Female sex	1.39 (1.051.84)	0.021	1.40 (1.011.93)	0.044
Diabetes	1.88 (1.352.60)	**<0.001**	1.34 (0.951.90)	0.097
Hypertension	2.20 (1.533.17)	**<0.001**	1.28 (0.861.90)	0.226
IHD	1.94 (1.422.66)	**<0.001**	1.20 (0.851.68)	0.299
ESRD	2.51 (1.773.55)	**<0.001**	1.90 (1.302.77)	**0.001**

*CI*confidence interval, *HR*hazard ratio, *IHD*Ischaemic heart disease, *ESRD*end-stage renal disease. Statistically significant associations are shown in bold text

^a^Results are adjusted for age, sex, Aboriginal and Torres Strait Islander status, IHD, diabetes, ESRD and hypertension

## Discussion

Recent systematic reviews have reported a decline in the incidence of major amputation in various countries and attributed this to improved multi-disciplinary care of the high-risk foot [1, 3] This finding is consistent with recent analyses of data from the Australian population [4, 20, 22, 23]. The age- and sex-adjusted incidence rates of major amputations in Queensland were reported to decrease by 26% in the general population and 45% in people with diabetes between 2005 to 2010 [4]. Similarly, Kurowski and colleagues reported a 6% annual decline in major amputation rates in people that had diabetes in Western Australia from 2000 to 2010 [23]. The Fremantle Diabetes Study (FDS) reported a 72% reduced risk of lower limb amputation in two cohorts with type II diabetes from the same Australian community over a 15-year period [22]. In the current study the estimated annual incidence of major amputation did not significantly change during the period from 2000 to 2015 and was higher than reported Australian national incidence rates for the available time period [20]. Reasons for this contrasting trend have not been identified in this study. It may reflect a growing burden of risk factors for major amputations in the North Queensland population [11, 13]. It may also be related to poor implementation of optimal medical and foot care management [24, 25].

Direct comparisons of major amputation rates between other studies are difficult since there is considerable variation in the methods used in previous investigations [1, 20]. A number of previous investigations have reported the cumulative incidence of major amputation or compared the amputation rates at two time points to illustrate the change in major amputations rates over time [1]. These studies have used a fixed at-risk population denominator throughout the study period which was obtained from census data [1, 9, 26]. Interpretation of these prior analyses are difficult as they did not account for population growth and did not capture trends in amputation incidence over time [1]. In the current study sensitivity analyses were performed to allow for various possible rates of population growth. When using a fixed at-risk population size a significant but small increase in major amputation incidence over time was observed. When an annual population growth rate of 2% was assumed, which is representative of the average per annum population growth rate for Townsville [19], no significant change in amputation rate over time was observed.

The clinical characteristics of people undergoing major amputation in this study were comparable to other Australian reports [9, 11]. Aboriginal and Torres Strait Islander people who underwent a major amputation were more likely to be of younger age, have diabetes and be female than non-Indigenous patients, consistent with some previous reports from regional populations in Australia and internationally [11, 27]. They were also more likely to have a diagnosis of ESRD, which is consistent with a previous study in North Queensland [28]. After adjusting for age and sex differences, Aboriginal and Torres Strait Islander people were approximately twice as likely, as non-indigenous people, to die during follow-up. After adjusting for differences in the prevalence of IHD, diabetes, ESRD and hypertension this increased risk of mortality disappeared. Individuals with ESRD had approximately 2-fold greater risk of mortality. The findings suggest that the increased mortality rate in Aboriginal and Torres Strait Islander people was likely because of their high rate of co-morbidities, particularly ESRD [28]. This observation has also been identified in other populations [29].

This study had several limitations including its retrospective design and relatively small sample size. The study used participants self-identifying as Aboriginal people and Torres Strait Islanders at the time of admission. This was not cross-referenced with any other records and it is therefore possible that the number of Aboriginal people and Torres Strait Islanders may have been under or over estimated. Patient data were obtained retrospectively from hospital records and data on smoking were not recorded due to inconsistent reporting of smoking behaviour in hospital charts. This is a recognised limitation of data from hospital records and has been highlighted in previous datasets [30]. Higher quality data is required to better assess the prevalence of risk factors for lower limb amputations in both Aboriginal people and Torres Strait Islanders and non-Indigenous Australians. Comparisons of major amputation rates with other populations was difficult due to the marked heterogeneity in reporting of these data in previous studies [1, 3]. Furthermore, comparisons with national major amputation rates were limited to a 4 year period [20]. Importantly, estimates of the number of Aboriginal people and Torres Strait Islanders within the population and the prevalence of diabetes within the THHS catchment area were based upon data collected at single time points. We were unable to include ESRD in the quasipoisson regression analysis as state-wide ESRD prevalence data stratified by each of the other covariates were not available to estimate the at-risk population. Notably, Aboriginal and Torres Strait islander status was not reported in 12,800 individuals from the census and this is a recognised limitation of census data [5]. Sensitivity analyses conducted suggested that the main findings presented in this paper were robust to the assumptions about the size of the background at-risk population. Data were not collected on the in-patient management of the included people, such as the surgical treatment of PAD. A previous study performed in the USA reported that African American patients with PAD had fewer revascularisation attempts and worse limb salvage rates than non-African American patients [31]. The higher major amputation rates in Aboriginal and Torres Strait Islander people could reflect less suitability for surgical treatment of limb ischemia but this was not investigated in this study [11, 13]. Diabetes and ESRD are risk factors for infra-popliteal artery disease, which has been associated with a higher risk of major amputation than more proximal athero-thrombosis [20]. In the current study, Aboriginal and Torres Strait Islander people had a greater prevalence of diabetes and ESRD and it is possible that they more frequently had infra-popliteal artery disease than non-Indigenous patients [13], although this was not assessed.

In conclusion, this study suggests that the annual incidence of major amputation in North Queensland is high and is not decreasing over time, which is in contrast with reports of reducing amputation rates in other regions within Australia [4, 22, 23] and internationally [1, 3]. Aboriginal and Torres Strait Islander people more frequently underwent major amputation compared to non-Indigenous people, highlighting a major health gap for this population. Furthermore, Aboriginal and Torres Strait Islander people undergoing major amputations were at a greater risk of subsequent mortality associated with high rates of co-morbidities, such as ESRD, in this population. Dissemination of effective preventative treatments is needed to reduce the high incidence of major amputation in North Queensland.

## Supplementary Information

**Additional file 1: Supplementary Table 1.** Quasipoisson regression analysis examiningtheassociation between Aboriginal and Torres Strait Islander status, sex, diabetes status, age and time on major amputation rates for varying assumptions aboutthe classification of Aboriginal and Torres-Strait Islander and non-Indigenous peoplein the Townsville census data. **SupplementaryTable 2.** Quasipoissonregressionanalysis examiningthe associationbetween time and major amputation rates for varying assumptions about thegrowth rate. **Supplementary Figure 1.** Kaplan-Meiercurve illustrating the cumulative proportion of Aboriginal and Torres Strait Islander andnon-Indigenous patients who had a contralateral major amputation.

## Data Availability

Requests for data should be directed to the corresponding author.

## Abbreviations

PAD: Peripheral arterydisease
THHS: The Townsville Hospital and Health Service
AKA: Above the kneeamputation
BKA: Below the knee amputation
HQA: Hindquarter amputation
ORMIS: Operating rooms management system
IHD: Ischaemic heart disease
ESRD: End-stage renal disease
ABI: Ankle-brachial index
IRR: Incidence rate ratios
AVA: Australasian Vascular Audit
FDS: Fremantle Diabetes Study
