# Supplementary material for: Major amputation rates and outcomes for Aboriginal and Torres Strait Islander and non-Indigenous people in North Queensland Australia between 2000 and 2015
Source: BMC Endocr Disord. 2021 May 21;21:101. doi: 10.1186/s12902-021-00764-z (PMC8139111; doi:10.1186/s12902-021-00764-z)
Supplement: Supplementary file 1 — Additional file 1: Supplementary Table 1. Quasipoisson regression analysis examiningtheassociation between Aboriginal and Torres Strait Islander status, sex, diabetes status, age and time on major amputation rates for varying assumptions aboutthe classification of Aboriginal and Torres-Strait Islander and non-Indigenous peoplein the Townsville census data. SupplementaryTable 2. Quasipoissonregressionanalysis examiningthe associationbetween time and major amputation rates for varying assumptions about thegrowth rate. Supplementary Figure 1. Kaplan-Meiercurve illustrating the cumulative proportion of Aboriginal and Torres Strait Islander andnon-Indigenous patients who had a contralateral major amputation. [file 12902_2021_764_MOESM1_ESM.docx]

**Major amputation rates and outcomes for Aboriginal and Torres Strait Islander and non-Indigenous people in North Queensland Australia between 2000 and 2015**

Tejas P. Singh MBBS MPH, Joseph V. Moxon PhD, Michael T. Meehan PhD, Rhondda E. Jones BSc (Hons) PhD, Yvonne Cadet-James, Jonathan Golledge MChir FRACS

**Supplementary Table 1.** Quasipoisson regression analysis examining the association between Aboriginal and Torres Strait Islander status, sex, diabetes status, age and time on major amputation rates for varying assumptions about the classification of Aboriginal and Torres-Strait Islander and non-Indigenous people in the Townsville census data.

**Supplementary Table 2.** Quasipoisson regression analysis examining the association between time and major amputation rates for varying assumptions about the growth rate.

**Supplementary Figure 1.** Kaplan-Meier curve illustrating the cumulative proportion of Aboriginal and Torres Strait Islander and non-Indigenous patients who had a contralateral major amputation.

**Supplementary Table 1.** Quasipoisson regression analysis examining the association between Aboriginal and Torres Strait Islander status, sex, diabetes status, age and time on major amputation rates for varying assumptions about the classification of Aboriginal and Torres-Strait Islander and non-Indigenous people in the Townsville census data.

| **Characteristic** |  | **Incidence Rate Ratio** |  |
| --- | --- | --- | --- |
|  |  | **(95% CI)** |  |
|  | **Baseline** | **Scenario I** | **Scenario II** |
| Aboriginal and Torres Strait Islander people | 2.75  (1.91-3.86) | 2.94  (2.04-4.12) | 2.29  (1.59-3.22) |
|  |  |  |  |
| Diabetes | 12.77  (9.69-16.89) | 12.77  (9.69-16.89) | 12.77  (9.69-16.88) |
|  |  |  |  |
| Male sex | 2.00  (1.54-2.61) | 2.00  (1.54-2.61) | 2.02  (1.55-2.66) |
| Age 0-34 years | Reference | Reference | Reference |
|  |  |  |  |
| Age 35-44 years | 3.51  (1.82-6.86) | 3.52  (1.83-6.89) | 3.53  (1.83-6.90) |
|  |  |  |  |
| Age 45-54 years | 5.35  (2.99-9.97) | 5.37  (3.00-10.01) | 5.38  (3.01-10.03) |
|  |  |  |  |
| Age >55 years | 11.57  (6.96-20.48) | 11.62  (6.99-20.57) | 11.60  (6.98-20.54) |
|  |  |  |  |

For binary variables, patients that did not have the risk factor were used as the reference group. Annual population growth was not incorporated in the sensitivity analyses. Baseline: Unspecified individuals from census were proportionately reclassified as Aboriginal people and Torres Strait Islanders and non-Indigenous (as described in the main article). Scenario I: Unspecified individuals from census were reassigned as non-Indigenous. Scenario II: Aboriginal and Torres Strait Islander population inflated by 20%, unspecified individuals from census were removed.

**Supplementary Table 2.** Quasipoisson regression analysis examining the association between time and major amputation rates for varying assumptions about the growth rate.

|  |  | Incidence Rate Ratio  (95% CI) | | | |  |
| --- | --- | --- | --- | --- | --- | --- |
|  |  |  |  |  |  |  |
| Annual growth rate | **0%** | **1%** | **1.5%** | **2%** | **5%** | **10%** |
| Calendar year | 1.04  (1.01 – 1.07) | 1.03  (1.00 – 1.06) | 1.02  (0.99 – 1.05) | 1.02  (0.99 – 1.05) | 0.99  (0.96 – 1.02) | 0.94  (0.92 – 0.97) |

For lower annual growth rates (0 or 1%) the model predicted a small but significant increase in the annual incidence of major amputations with time. For annual growth rates between 1.5 and 5% the time trend was no longer significant, whilst for growth rates exceeding 5% the time trend was reversed (i.e. negative). The main analysis assumed a 2% annual growth rate with calendar year. Reported growth rates were applied to the total cohort.

**Supplementary Figure 1.** Kaplan-Meier curve illustrating the cumulative proportion of Aboriginal and Torres Strait Islander and non-Indigenous patients who had a contralateral major amputation.

Indigenous, Aboriginal and Torres Strait Islander people. Differences between both groups compared using the log-rank test (*p*=0.432). The green line represents patients who are non-Indigenous, and the pink line represents Aboriginal and Torres Strait Islander patients. Vertical lines represent participants who were censored during follow-up.
